# Social Cognition—A Successful First Year

**DOI:** 10.3389/fpsyt.2018.00719

**Published:** 2018-12-19

**Authors:** Sören Krach

**Affiliations:** Psychiatry and Psychotherapy, University of Lübeck, Lübeck, Germany

**Keywords:** Tourette, echophenomena, psychophysiology, psychiatry, social cognition

*Frontiers in Psychiatry—Social Cognition* was launched in 2018, with the goal of providing an Open Access venue for high quality research. Research on social cognition in psychiatry has seen rapid growth in the past 5 to 10 years and the community's reaction to the launch of the section reflected this need for a new specific publishing outlet. As a journal with an Impact Factor of 2.857 and an excellent reputation, *Frontiers in Psychiatry*'s *Social Cognition* section has received a high level of interest from both key opinion leaders, as well as rising stars in the research field. We are very proud that our journal has stimulated such interest in its launch year and we hope that this will continue into 2019 and beyond.

As a new section our main task in 2018 was to recruit internationally highly-recognized scholars in the field of social cognition in psychiatry spanning very broad research topics. Currently, our new section involves 13 Associate Editors from Europe, Asia, and South & North America as well as 58 review editors from all over the world with expertise in social learning, reward processing, decision-making, or social interaction. Our Associate Editors have backgrounds in computational modeling, structural, and functional brain imaging techniques, psychophysiology, experimental design, virtual reality, brain stimulation, or pharmacological interventions. We had a great start to the year in terms of submissions, with already three very innovative and promising Research Topics being launched. The first article collection, led by Simon Surguladze, addresses the question of Empathy in a Broader Context: Development, Mechanisms, Remediation and has received over 1,000 views with no articles published yet. A second Research Topic was launched on the hotly debated topic on the effects of neuromodulators on social learning and decision-making, led by Andreea Diaconescu (Pharmacological Interventions Targeting Social Motivation and Social Reward). Finally, The Social Side of Gilles-de-la-Tourette Syndrome targets the question of how echophenomena, coprolalia, or other socially inappropriate behavior in neurological and psychiatric conditions have their environmental determinants and neural, cognitive, and affective underpinnings. We expect several other high-profile Research Topics to be submitted early in 2019. Our clinical focus is on major psychiatric conditions such as schizophrenia, depression, addiction, or anxiety, but we also welcome less-investigated etiologies such as e.g., Tourette-Syndrome.

Finally, we would like to thank all our supporters, our Associate Editors, Review Editors and Authors. Without their continued enthusiasm, commitment and support, Social Cognition may not have had such a fantastic first year.

## Author Contributions

The author confirms being the sole contributor of this work and has approved it for publication.

### Conflict of Interest Statement

The author declares that the research was conducted in the absence of any commercial or financial relationships that could be construed as a potential conflict of interest.

